# A reduced-scale canyon street to study tree climate benefits: summer 2020 data with well-watered apple trees

**DOI:** 10.1038/s41597-024-03650-0

**Published:** 2024-09-18

**Authors:** Sophie Herpin, Souleymane Mballo, Melvin Manteau, Dominique Lemesle, Agathe Boukouya, Bénédicte Dubuc, Lydie Ledroit, Patrice Cannavo, Sabine Demotes-Mainard, Pierre-Emmanuel Bournet

**Affiliations:** 1 Institut Agro, EPHOR, 49045 Angers, France; 2 IRSTV, FR CNRS 2488, 44321 Nantes, Cedex 3 France; 3 Univ Angers, Institut Agro, INRAE, IRHS, SFR QUASAV, F-49000 Angers, France

**Keywords:** Atmospheric science, Environmental impact

## Abstract

An outdoor reduced-scale canyon street was set-up in Angers, France, to study the impact of well-watered trees on urban microclimate and human comfort, with an integrated approach of the soil-plant-atmosphere continuum. Data were acquired during 26 days in summer 2020. The street is oriented north-south, with an aspect ratio of 1. It is organized in three zones: two zones with a central alignment of 5 ornamental apple trees, and one zone without trees. The water inputs are controlled through a drip-irrigation system. Each zone is instrumented to characterise the local microclimate and energy fluxes, the soil water status, and tree leaf temperature. To allow a better understanding of the physical mechanisms at stake in tree services, tree transpiration as well as crown light interception are also quantified, and the trees are characterised in terms of leaf area and crown dimensions. The data can benefit to researchers in urban meteorology and environmental physics. It can also provide reference data to run and evaluate microclimate models, especially regarding plant-atmosphere interactions.

## Background & Summary

Soil artificialization in urban areas has a direct impact on cities energy budget^1^: evapotranspiration from vegetation is reduced, while net radiation and energy storage by construction materials are enhanced. As a result, cities experience urban overheating, which can be critical for thermal comfort or even physical health of inhabitants during summer time. In a context of global climate change where the frequency and intensity of heat waves are very likely to increase^2^, it is paramount to look for adaptation solutions in urban areas. Street trees are an interesting solution, as they can provide cooling benefits through both shading and evapotranspiration, and can be implemented in a large number of streets, close to inhabitants. However, their benefits largely depend on the urban configuration (street orientation and aspect ratio), urban materials, meteorological conditions, and water availability in the soil, in addition to intrinsic parameters such as leaf area index (LAI), or stomatal regulation.

Street tree benefits have been previously addressed in some real-scale *in-situ* experiments, either at microscale^3–7^ (with meteorological stations at several fixed locations within a street) or at an intermediate scale^8–11^ (with mobile measurements in a neighbourhood, involving some spatial and temporal averaging along the street axis). In addition to air temperature, these studies have looked in particular at surface temperature, mean radiant temperature and/or radiation fluxes^7,9,10^, tree transpiration^3,5,6^, or thermal comfort indices^4,6^. Real-scale *in-situ* experiments are sometimes difficult to interpret or to transpose because the urban configuration is non canonical (for example, buildings are not of constant height or with the same wall colour along the street length), and the experiment often lacks an equivalent control zone, without trees. Reduced-scale experiments^12–14^ can overcome these issues, and, even if they cannot not fully reproduce the urban environment, they can be useful for quantifying physical phenomena and to validate numerical models. However, the availability of open reference experimental datasets on vegetated canyon street is clearly not sufficient: three relevant open-access dataset at street scale could be found from studies identified in the literature^15^ or from Environmental Data Initiative deposit, or dataset search engines (search rule on Google Dataset Search Engine (https://datasetsearch.research.google.com/): (vegetation OR tree OR plant) AND (urban OR street OR city) AND (climate OR temperature OR heat OR cooling))). The first dataset^16^ comprises air temperature, relative humidity and energy fluxes in Basel, Switzerland, supporting a research work on COSMO-BEP-Tree model^17^. The second dataset^18^ consist in an analysis of field measurements of air temperature from 77 global sites in 35 cities, obtained by digitizing from the global published literature, and supplemented with vegetation analysis (NVDI, LAI…) from teledetection gridded data. The dataset was used to analyse the effect of background climate and plant phenology on tree benefits^19^. The third dataset^20^ consist of bicycle-mounted mobile air temperature measurements in a mid-size city in the USA, with a range of tree canopy and impervious surface cover.

Microclimate models can simulate canonical geometries, with controlled meteorological conditions. Some integrated energy-balance models^9,21,22^ or microclimate models^7,23–25^ including vegetation schemes do provide partial validation of tree effect with experimental data^3,7,9,26,27^, but could benefit from more extensive open experimental datasets, including soil water moisture data and vegetation data (especially on trees), such as crown dimension, leaf area index, leaf surface temperature, radiation interception and transpiration. Indeed, experimental studies often address soil water status in separate studies^28,29^ from those addressing microclimate and thermal comfort benefits, which is a shortcoming to improve latent heat flux evaluation^30^.

In order to address this challenge, an outdoor reduced scale canyon street facility was constructed and instrumented over the soil-plant-atmosphere continuum in Angers, France. To evaluate tree climate benefits, the street has two treed zones and one non-vegetated control zone, with asphalt pavement. Tree benefits can thus be evaluated by comparing the microclimate inside the treed and the non-vegetated zones. Urban overheating can also be assessed by comparing the microclimate in the non-vegetated zone with the microclimate outside the street. The water inputs in the soil of the trees are controlled thanks to a dripping irrigation system and to a lid on the tree container preventing water inputs from rain. Some results of summer 2020 with well-watered ornamental apple tree have already been analyzed^13,31,32^, on specific days, and focusing on atmosphere variables. The present contribution proposes to extend the dataset to the soil-tree-atmosphere continuum over 26 consecutive days of summer 2020, with open access to the data. The dataset includes two ranges of tree crown size and leaf area, making it possible to study the impact of LAI and street coverage ratio on the cooling benefits.

## Methods

### Experimental site, facility and measurement period

**Fig. 1:**
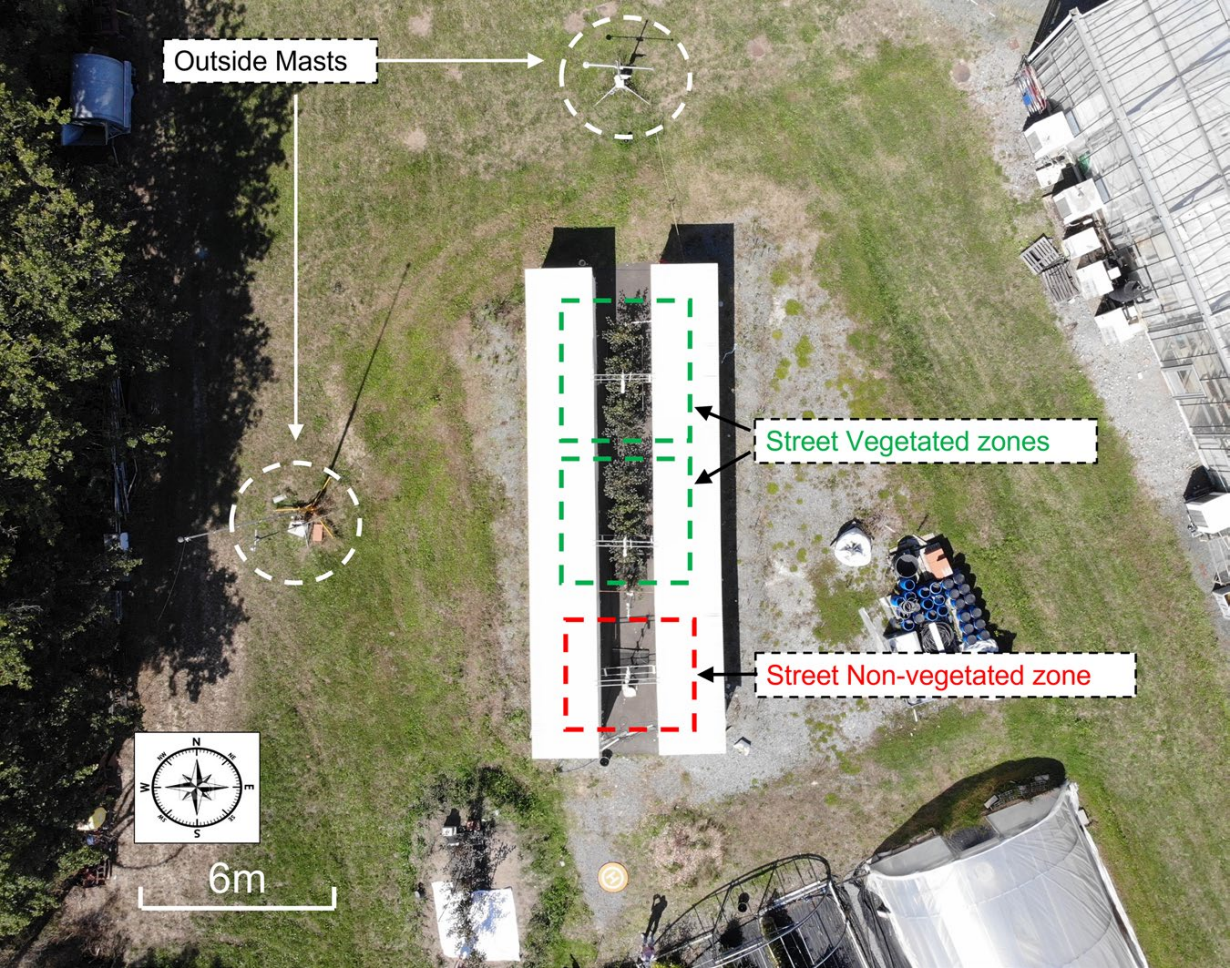
Top-view of the experimental site with the street and its environment, showing the two vegetated zones of the street, the non-vegetated zone of the street, and the position of the two outside meteorological masts.

**Fig. 2:**
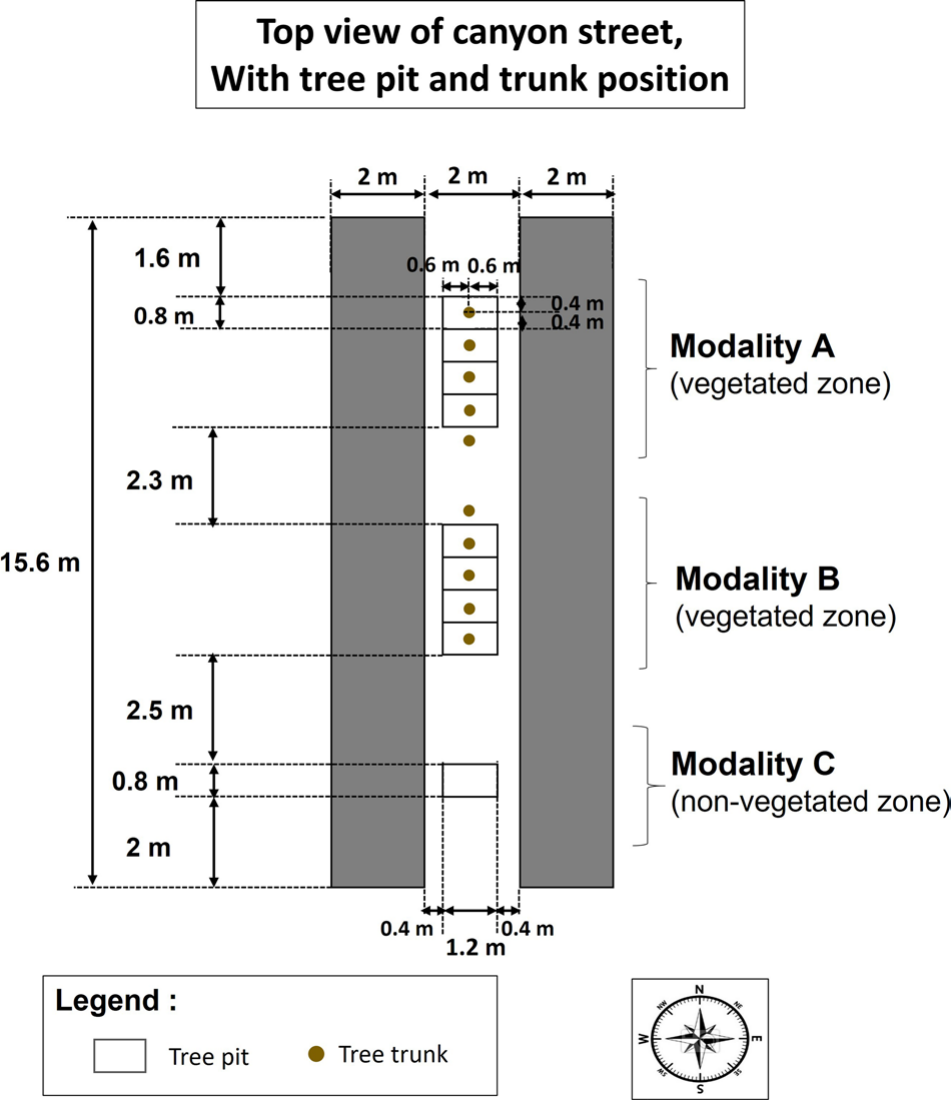
Top view of the street, with street dimensions, tree positions, and the 3 modalities investigated.

The experimental site is located in Angers, France (GPS coordinates: 47° 28′ 47″ N, 0° 36′ 33″ W), in a suburban environment, classified as “open low-rise” in the Local Climate Zone classification^33^. The climate is classified as Cfb (temperate oceanic) according to the Köppen-Geiger classification. The experimental facility consists of a single 1/5 scale canyon street, north-south oriented, which is 15.6 m long, 2 m wide, and flanked on both sides by 2 m high and wide buildings (Fig. 1). The street is organized in three zones (Figs. 1, 2): two zones with a central alignment of 5 trees each, in the northern part (modality A) and centre part (modality B) of the street, and one zone in the south of the street without trees (modality C). More details on the trees are given in the next section. The materials used for the street construction are typical urban materials. The floor of the street is constituted of a 0.04 m thick layer of asphalt followed in depth by a 0.25 m thick layer of gravel. The building walls towards the street are covered with a white plaster, and made of 0.1 m thick concrete insulated with a 0.12 m thick layer of expanded polystyrene on their inner side. The roof and remaining walls are made with wood beams and 12 mm thick OSB wood panels, protected by an HPV humidity barrier and covered with a 0.75 mm thick white steel pan to ensure building waterproofness (see Fig. 6).

**Fig. 3:**
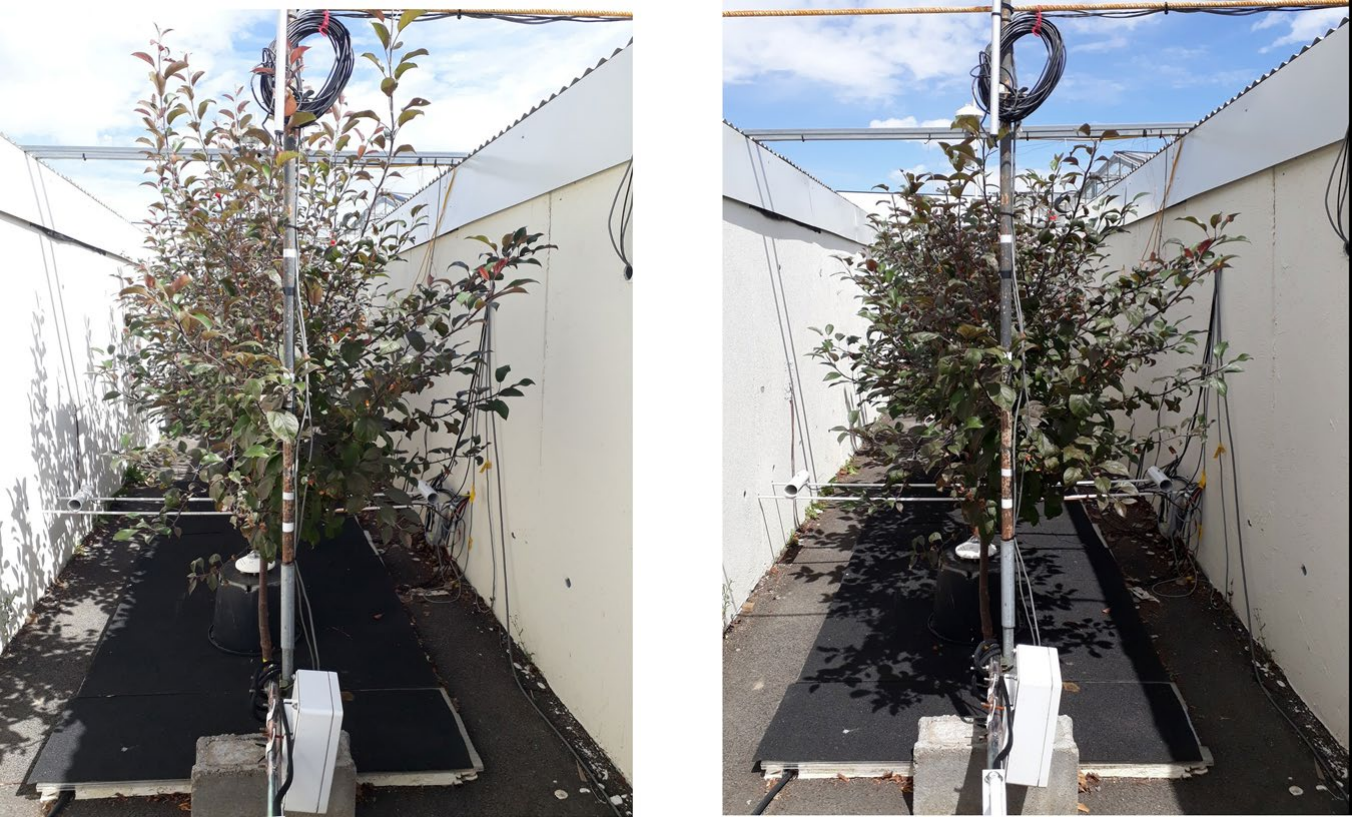
Photo of the trees in the canyon street, taken from the south of the street, on 2020/07/23 at 09:30 UTC just before trimming (left) and on 2020/07/23 at 11:15 UTC just after trimming (right).

The trees in modalities A and B are crab apple trees (*Malus coccinella*® ‘Courtarou’), all obtained from cuttings in the same nursery in Angers, France, and grown thereafter in the same conditions. The trees were pruned in winter 2018–2019, transplanted in the street in March 2019, and then pruned again in winter 2019–2020 so as to obtain the development of a crown at the desirable height proportionally to the street. At the time of the experiment (summer 2020), the trees were 3-years old. Inside the street, the trees were planted in 80 L containers placed inside individual pits. The substrate used in the containers was made of a 0.25 m thick mixture of top-soil/compost in a 60/40% volume ratio, over a 0.1 m thick mixture of topsoil-compost/stone in a 65/35% volume ratio. The final volume of top-soil/compost was 44.5 L/tree. To control soil water content, the trees were irrigated with drippers, and the pits and containers were covered with a lid to prevent rainwater to enter the pits. At the bottom of the containers, underneath the substrate, a concrete slope guided drainage water to an external recipient (see Fig. 7). The experimental measurement period covers 26 days, from 07/10/2020 to 04/08/2020, and, during the experiment, on July 23rd 2020, the crowns of the trees were trimmed at a height z = 1.7 m above ground level and at a width of 1.2 m with the aim to limit their development within the street dimensions. Due to this trimming, the tree characteristics underwent a discontinuity on July 23rd (23/07/2020), the crown dimensions and leaf area being reduced at that time. A photo of the trees before and after trimming is provided in Fig. 3. The tree characteristics can thus be established over two separate periods: one period strictly before the day of trimming, and another strictly after the day of trimming. This also makes it possible to benefit from two datasets with different tree characteristics. All microclimate, soil, leaf temperature, and PAR radiation data presented in this section were measured every 10 seconds, averaged and then recorded over 10 min periods. The present dataset is therefore given with a 10 min time step.

### Location of microclimate instrumentation

**Fig. 4:**
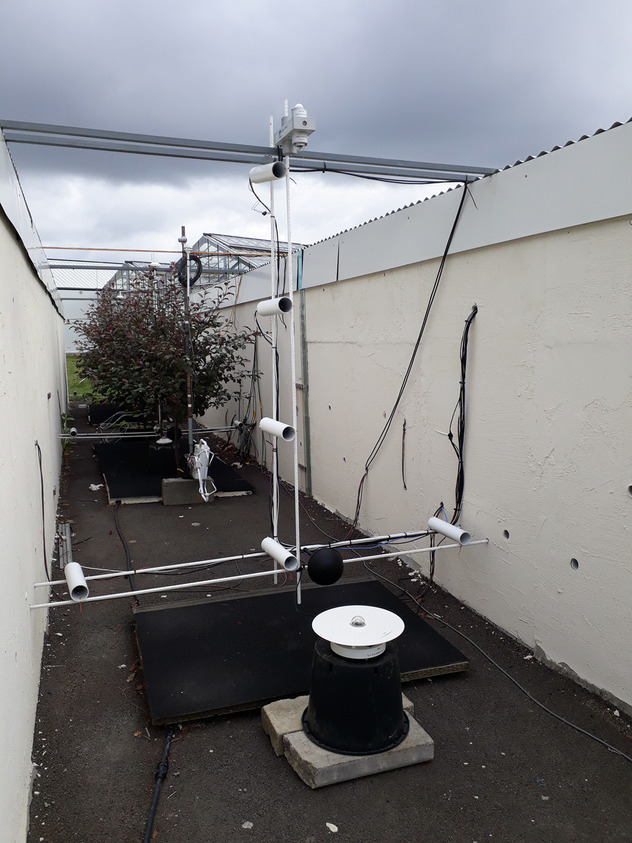
Microclimate instrumentation in non-vegetated modality (C) of the street (visible in the forefront: Temperature and humidity sensors in their mechanically ventilated shelter, black globe, pyranometer, 4 components radiometer); photo taken on 2020/07/02.

**Fig. 5:**
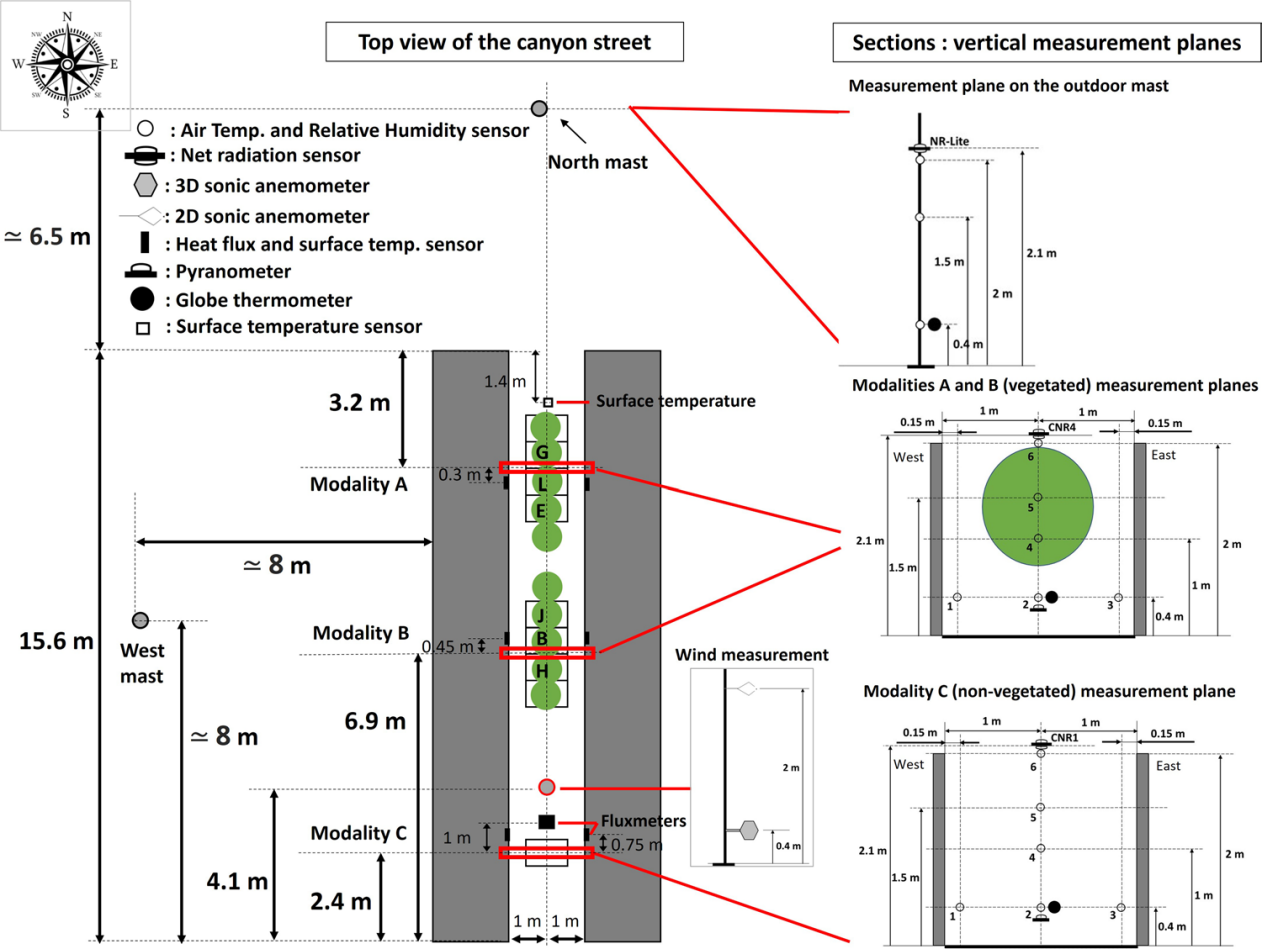
Description of microclimate measurements.

**Fig. 6:**
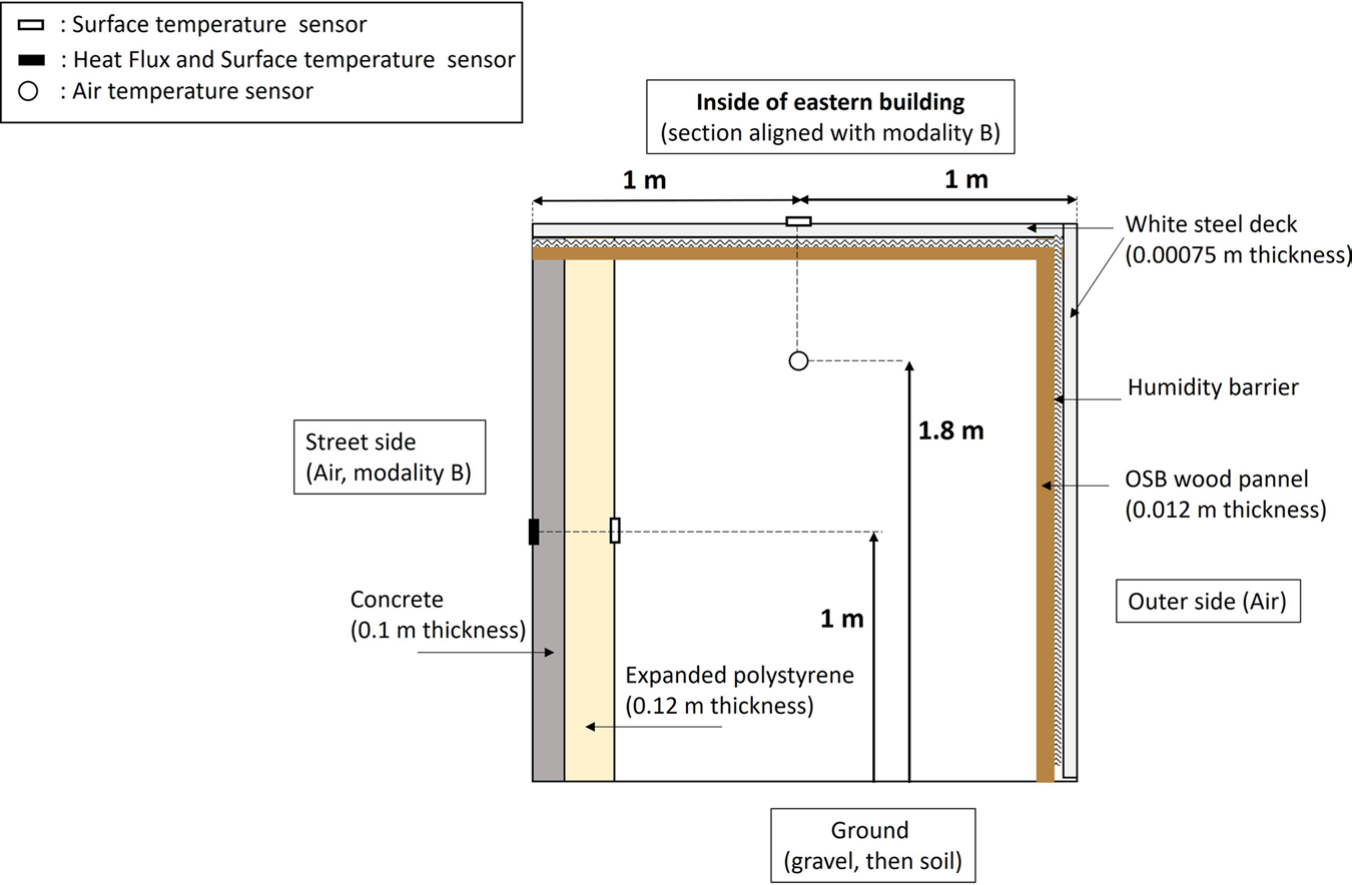
Instrumentation of the eastern building.

The street microclimate was monitored in each of the three investigated zones of the street i.e. in modalities A and B in the north and center part of the street, each with a 5-trees alignment, and in the non-vegetated (without trees) modality C in the south of street. Within each of these modalities, a vertical microclimate measurement plane was instrumented with a similar set of sensors (described in the next paragraphs, visible in Fig. 4 and in Fig. 5). The objective was to be able to compare the microclimate in the vegetated and non-vegetated modalities, in order to quantify the impact of trees on the street microclimate. Additionally, two meteorological masts were placed on-site, above grassy ground outside the street on its west side at 8 m from the western building and north side, at 6.5 m from the street (Figs. 1, 5), to collect meteorological conditions in the vicinity of the street. The data collected on the masts enable to quantify the incident conditions on the street, can provide input climatic data in a prospect of modeling, and provide reference data to analyze the effect of the street on the microclimate. A photo of microclimate instrumentation in the non-vegetated modality is provided as an example in Fig. 4. An overview of overall instrumentation is given in Fig. 5. Finally, several measurements were also operated inside the eastern building (Fig. 6). The characteristics of the microclimate sensors are given in Tables 1, 2.

**Table 1:** Characteristics of microclimate sensors for air, globe and surface temperatures, air relative humidity, surface heat flux and wind speed and direction (n.c. means: not communicated from manufacturer).

Measured variables	Sensor type	Measurement range	Accuracy	Reference, manufacturer
**Air temperature** (street: vegetated modalities A and B, north mast)	Platinum probe (in mechanically ventilated shelter)	−40 to 80 °C	±0.2 °C	Pt1000 HMP110, Vaisala
**Air temperature** (street: non-vegetated modality C)	Platinum probe (in mechanically ventilated shelter)	−70 to 180 °C	±0.2 °C at 20 °C	Pt100 HMP337, Vaisala
**Air temperature** (inside the building)	Platinum probe	−50 to 200 °C	±0.15 °C + 0.002 * [T°C]	PT100 RS class A
**Relative humidity** (street: vegetated modalities A and B, north mast)	Capacitive probe (in mechanically ventilated shelter)	0 to 100%	±1.5% for RH ≤ 90%	HMP110, Vaisala
**Relative humidity** (street: non-vegetated modality C)	Capacitive probe (in mechanically ventilated shelter)	0 to 100%	±(1.0 + 0.008 × indicated value) % RH	HMP337, Vaisala
**Globe temperature** (street and north mast)	Platinum probe (in 15 cm diameter black globe)	−50 to 200 °C	±0.15 °C + 0.002 * [T°C]	PT100 RS class A
**Surface temperature** (inside the building)	Flat platinum probe	−50 to 200 °C	±0.15 °C + 0.002 * [T°C]	PT100 RS class A
**Surface temperature** (street: walls in all modalities and ground in non-vegetated modality C)	Fluxmeter	−30 to 70 °C	n.c.	Captec
**Surface temperature** (street: ground in vegetated modality A, building roof-outerside; building wall – inner side)	Flat platinum probe	−50 to 150 °C	±0.3 °C + 0.005 * [T°C]	Pt100 TCSA class B
**Surface Heat fluxes** (street: walls and ground)	Fluxmeter	Up to 150 kW	±3%	Captec
**Wind speed & wind direction** (street: z = 0.4 m)	3D sonic anemometer	0 to 30 m s^−1^ 0–359°	±0.08 m s^−1^ ± 0.7° à 1 m s^−1^	CSAT3, Campbell Scientific Ltd
**Wind speed & wind direction** (west mast, z = 10 m)	2D sonic anemometer	0 to 60 m s^−1^ 0–359°	±2% à 12 m s^−1^ ± 3° à 12 m s^−1^	Wind Sonic, Gill instrument
**Wind speed & wind direction** (west mast: z = 2 m and z = 5 m; street: z = 2 m)	2D sonic anemometer	0 to 40 m s^−1^	±0.12 m s^−1^ ± 1.5°	CV7, LCJ

**Table 2:** Characteristics of microclimate sensors for radiation fluxes (n.c. means: not communicated from manufacturer).

Measured variables	Sensor type	Measurement range	Accuracy	Reference, manufacturer
**Radiation**, (street: vegetated modalities A and B, z = 2.1 m):	4 component radiometer			CNR4, Kipp & Zonen,
Short wavelength	Pyranometer	up to 2000 W/m^2^	±5% on daily totals	
Long wavelength	Pyrgeometer	n.c.	±10% on daily totals	
**Radiation**, (street: non-vegetated modality C, z = 2.1 m):	4 component radiometer			CNR1, Kipp & Zonen,
Short wavelength	Pyranometer	up to 2000 W/m^2^	±10% on daily totals	
Long wavelength	Pyrgeometer	n.c.	±10% on daily totals	
**Radiation**, short wavelength (street: modality A at z = 0.4 m; north mast)	Pyranometer	n.c.	Accuracy 1% in spectral band in nm: 300–2500	CE180, CIMEL
**Radiation**, short wavelength (street: modality B at z = 0.4 m)	Pyranometer	up to 4000 W/m^2^	±3% in daily and hourly totals	CM11, Kipp & Zonen
**Radiation**, short wavelength (street: modality C at z = 0.4 m)	Pyranometer	up to 2000 W/m^2^	±10% in daily totals	CM3, Kipp & Zonen
**Radiation**, net total (North mast)	Net radiometer	n.c.	n.c.	NR-Lite, Kipp & Zonen

### Air Temperature and Relative Humidity

Air temperature (Ta) and relative humidity (RH) were measured in the street in each modality using Vaisala HMP sensors (±0.2 °C and ±1.5% for RH in [0%;90%]) at four heights (0.4 m, 1 m, 1.5 m and 2 m from the ground) on a vertical axis at equal distance from the two walls. In each plane, two additional measurement points were taken at 0.4 m high, at 0.15 m distance from each wall. On the north mast outside the street, temperature and relative humidity were measured at three heights from the ground: 0.4 m, 1.5 m and 2 m. These different heights were chosen so that 0.4 m in the scale facility corresponds to 2 m height at full scale (for thermal comfort evaluation), 1 m corresponds to half the height of the street walls, 1.5 m is inside tree crown and 2 m correspond to the top of the street walls. These sensors were protected from direct shortwave radiation using a white-painted cylindrical shelter, mechanically ventilated. It should be noted that the air temperature measurements at z = 0.4 m from the ground can be located inside the thermal boundary layer developing on the surface (depending on the insolation and wind conditions of the day), and therefore be affected by surface temperature. The effect is evaluated as very low in the vegetated zones (because the ground is shaded by the trees, and does not heat up), but can be significant in the non-vegetated zone of the street (+1.0 °C, evaluated on 2020-07-29^13^ (sunny day and low wind) at solar noon between air temperature at 0.4 m and 1 m from the ground, for example). In the eastern building, air temperature was measured at 1.8 m from the ground, in the middle of the building, in front of modality B, using a Pt-100 class A sensor.

### Radiation data

In each street modality, at 2.1 m from the ground, just above building height, a 4-components radiometer (Kipp & Zonen CNR) was used to measure downward and upward short-wave and long-wave radiation fluxes. In vegetated modalities A and B, the upper branches of the trees were strictly below this sensor from the 24/07/2020 to the 04/08/2020 (after trimming), so the sensor was fully exposed to the sun at that period. In contrast, from the 07/07/2020 to the 22/07/2020 (before trimming), the upper branches may have reached the sensor, and the 2.1 m high sensors may then be partially shaded by leaves and branches. In order to retrieve the incident radiation over the whole period without any shading on the sensor, it is therefore recommended to use the radiometer in modality C which is non-vegetated. At 0.4 m from the ground, a pyranometer was also used to measure the received or transmitted downward short-wave radiation, both in the non-vegetated zone (modality C) and under the tree crowns (modalities A and B). Outside the street, a net radiometer (Kipp & Zonen NR-Lite) was used at 2 m from the ground to measure net radiation, and a pyranometer was used at the same height to measure reflected short-wave radiation from the ground (North mast). Globe temperature was measured at 0.4 m from the ground, both inside the street (in each modality) and outside the street (on the north mast) using a Pt100 sensor class A in a 15 cm diameter copper black-painted sphere.

### Surface temperature and conductive heat fluxes

Surface temperature and conductive heat fluxes were measured on the eastern and western street walls, at 1 m from the ground in each measurement plane, using Captec fluxmeters (±3% accuracy on heat flux), and also at 2 cm depth in the asphalt ground in modality C of the street. Surface temperature on the asphalt ground was also measured in modality A, a few centimeters north of the tree pit at the northern end of the modality, using a Pt-100 sensor class B. Finally, the roof outer surface temperatures of the eastern and western buildings were also measured in front of modality B, in the middle of the roof using Pt-100 sensors class B. Inside the eastern building, the wall inner surface temperature on the expanded polystyrene in front of modality B at 1 m height from the ground was measured using the same type of sensor. The position of the sensors outside and inside the Eastern building is represented in Fig. 6.

### Wind speed and direction

Inside the street, wind speed and direction were taken between modality B and C at two heights, 0.4 m and 2 m from the ground, using a CSAT3 sonic anemometer (±0.08 m/s) measuring the 3 components of velocity and a LCJ CV7 2D sonic anemometer (±0.12 m/s) measuring the 2 horizontal components of velocity, respectively. Outside the street, wind speed and direction were measured on the west mast, at three heights from the ground: 2 m, 5 m (using a LCJ CV7 2D sonic anemometer) and at 10 m (using a Mc Gill 2D wind sonic anemometer, ±2% à 12 m/s).

### Soil instrumentation

**Fig. 7:**
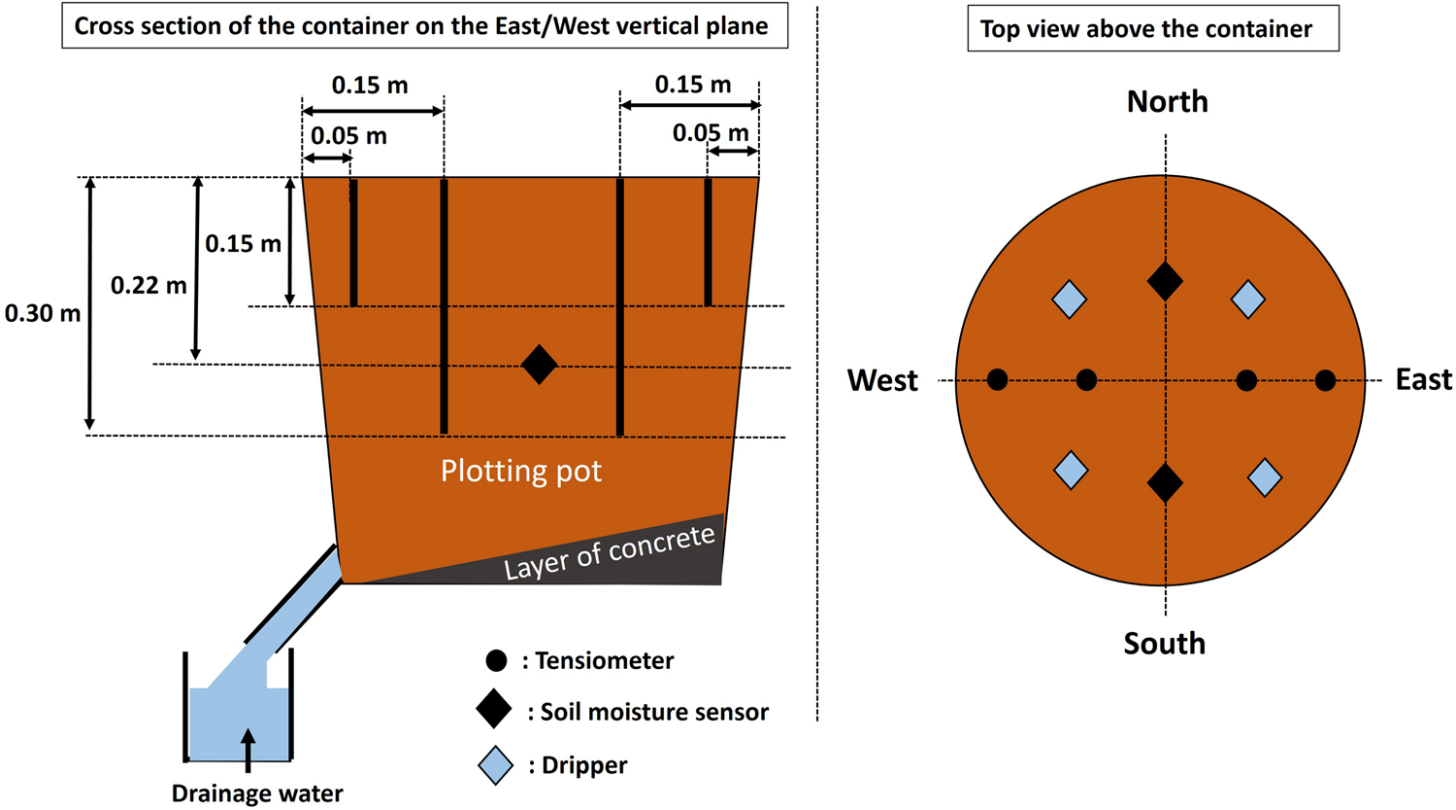
Description of the tree container and soil instrumentation.

Soil volumetric water content was measured in the soil of trees G and E in modality A, and of tree H in modality B at 0.22 m depth, with two capacitive sensors (Decagon EC-5, ±0.02 cm^3^ water/cm^3^ soil) per tree: one on the north side and one on the south side of the container. Soil matric potential was also measured in the soil of G, L and E trees in modality A, and B, H trees in modality B to monitor soil water availability, using tensiometers (SDEC STCP850) at 0.15 m and 0.30 m depth on the west and east side of each container (all mentioned trees were initially equipped at the same positions in their soil, but some soil sensors did not provide valid data and are therefore not reported in the dataset). A summary of soil instrumentation can be found in Fig. 7^13^, and the reader can also refer to Fig. 5 to link tree label and its position in the street. The sensors characteristics are given in Table 3.

**Table 3:** Characteristics of soil sensors.

Measured variables	Sensor type	Measurement range	Accuracy	Reference, manufacturer
Volumetric Water Content	Capacitive	0–1	±0.02 cm^3^ water/cm^3^ soil	EC-5 Decagon
Matric Potential	Tensiometer	−1000 hPa up to 5 hPa	Hysteresis: ±0.2% of full scale	STCP850, SDEC

### Tree crown instrumentation

Leaf surface temperature was measured on 4 leaves in tree L in modality A (1 leaf at 1 m from the ground on the west side, and 3 leaves at 1.5 m from the ground on the east side, center, and west side of the crown) and on 5 leaves in tree B in modality B, at equivalent positions plus 1 leaf at 1 m from the ground on the east side of the crown using thermocouples taped to the abaxial surface of the leaves with adhesive skin sutures (steri-strip 3 M, width 6 mm). This tape is made of microporous breathable material, and the taping surface occupied only a small portion of the leaf, roughly 10% of the abaxial surface. It is therefore expected that the impact of the tape on the leaf energy balance and surface temperature remained negligible. Indeed, no visual impact such as early yellowing of the instrumented leaves was noticed during the experiment. The adherence of the thermocouples to the leaves was checked every working day (approx. five times a week) and when a thermocouple was no longer in place the data since the previous check were flagged as non-representative.

Downward Photosynthetically Active Radiation (PAR) was measured using Solems PAR/LE sensors (±10% accuracy) (ca. 30 cm long) on the same trees (tree L in modality A and tree B in modality B) at 4 different heights from the ground: 2 m (i.e. within the tree crown from 10/07/2020 to 22/07/2020, and above the tree crown after trimming, from the 24/07/2020 to 04/08/2020), 1.5 m & 1.1 m (within the tree crown), and 0.65 m (below the tree crown).

**Fig. 8:**
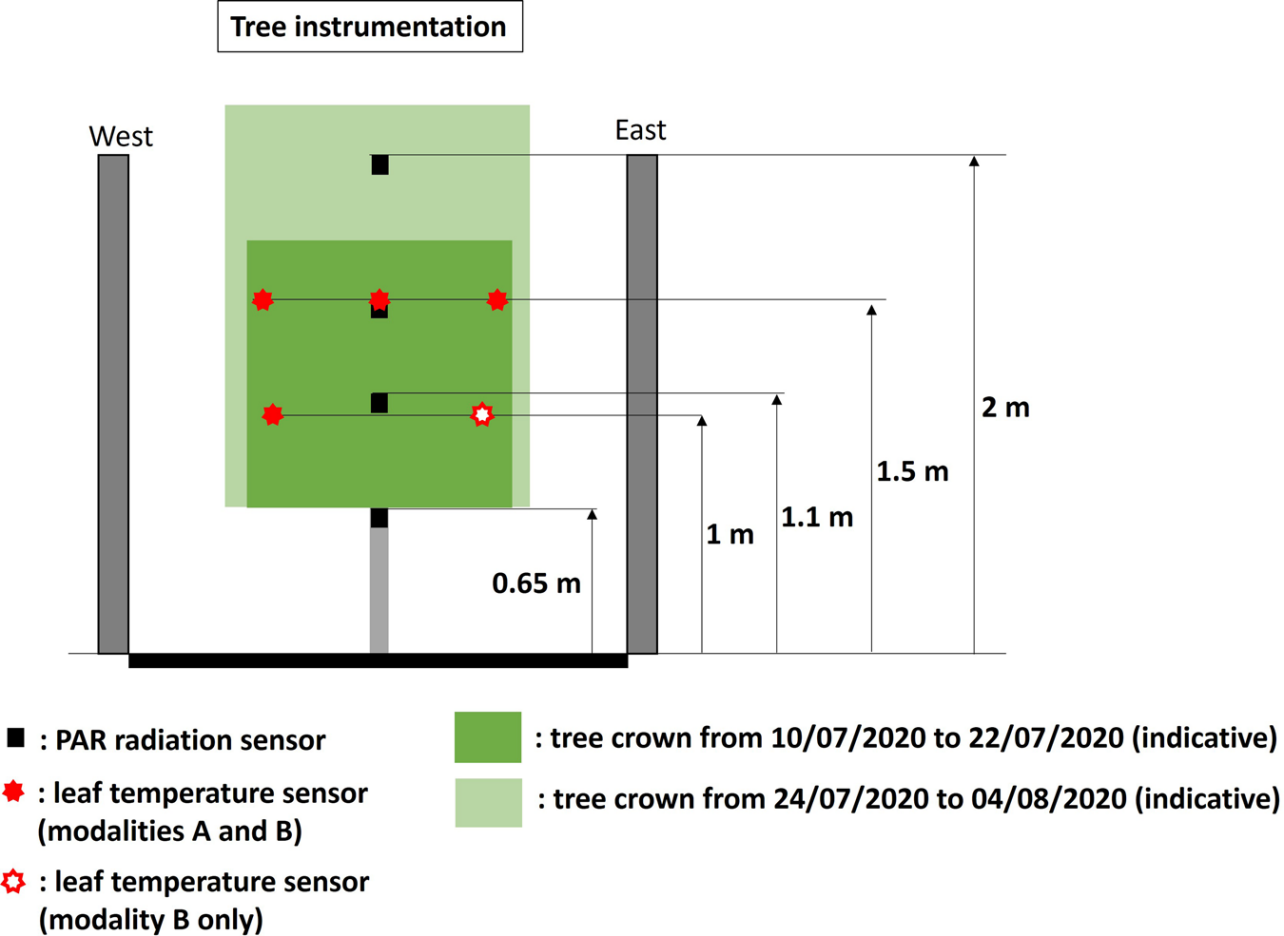
Tree Instrumentation in vegetated modalities.

A summary of tree instrumentation can be found Fig. 8, and the sensors characteristics are given in Table 4.

**Table 4:** Characteristics of tree sensors.

Measured variables	Sensor type	Measurement range	Accuracy	Reference, manufacturer
PAR radiation	Photosynthetically Active Radiation (PAR) sensor	0–544 W/m^2^	±10%	PAR/LE, Solems
Leaf surface temperature	T-type Thermocouple	−40 °C to 125 °C	±0,5 °C or 0.40%	RS IEC Thermocouple Cable - PFA Insulated Double Twisted Pair

### Evaluation of tree crown volume, leaf area, Leaf Area Index and Leaf Area Density

The tree morphological characteristics were evaluated over two sub-periods: from the 10/07/2020 to the 22/07/2020 and from the 24/08/2020 to the 04/08/2020, that is strictly before and after the tree trimming operation that occurred on the 23/07/2020.

The tree crown bounding volume was approximated to a rectangular parallelepiped, and its dimensions were measured on 17/07/2020 (before trimming), and considered to be valid over a period extending from one week before measurements (from 10/07/2020) until the day before trimming (22/07/20), which was less than one week after measurement. The length of the parallelepiped is determined by the distance between the trunks of the two trees at each end of the alignment, plus the average distances between the end of the two longest branches and the trunk for the tree to the north and south of the alignment, using a measuring tape. The height of the base of the parallelepiped is the average, on the three central trees, of the insertion height of the lowest branch and the lowest leafy point. The top height of the parallelepiped is the average height of the highest branches, minus 0.05 m on the three central trees; the width is the average of the maximum tree widths, minus 0.05 m on each side, on the three central trees; 0.05 m is subtracted when the measurement relies only on the most extreme branch of the trees. After trimming, the dimensions of the bounding volume were considered to be given by the trimming parameters (upper limit of the bounding volume set to z = 1.7 m and width of the bounding volume equal to 1.2 m). Since tree aerial development is usually stopped for 2 weeks after a trimming procedure, the crown dimensions remained constant from the day just after trimming (24/07/2020) until 04/08/2020, two weeks later.

The leaf area of the three central trees in each zone was measured in July, before trimming, with an allometric procedure. Measurement took 3 days (on the 9^th^, 15^th^ and 16^th^ of July). Apple trees display axes whose internodes elongate and axes that remain short; axes that were shorter than 5 cm were classified hereafter as short axes and the others as long axes^34^. On each tree the foliated length of all long axes was measured with a ruler and the number of foliated short axes were counted. In addition, four long and four short axes were randomly sampled on each central tree, the length and width of each of their leaves were measured with a ruler and the foliated length of the 4 long axes was measured.

Individual leaf area was calculated by allometry as:

1 $$\begin{array}{c}{\rm{A}}=0.7149\times {\rm{L}}\times {\rm{W}}\end{array}$$

with A the individual leaf area (cm²), L the leaf length (cm) and W the leaf width (cm), and the leaf area per sampled axis was calculated by summing the area of all its leaves.

Coefficients of leaf area for long axes (C_Long_) and short axes (C_Short_) were calculated at the scale of the axis and then averaged by tree, as:

2 $$\begin{array}{c}{{\rm{C}}}_{{\rm{Long}}}=\frac{{\rm{Leaf}}\,{\rm{area}}\,{\rm{per}}\,{\rm{axis}}}{{\rm{Foliated}}\,{\rm{length}}\,{\rm{of}}\,{\rm{the}}\,{\rm{axis}}},{\rm{in}}\,{{\rm{m}}}^{2}/{\rm{m}}\end{array}$$

3 $$\begin{array}{c}{{\rm{C}}}_{{\rm{short}}}={\rm{Leaf}}\,{\rm{area}}\,{\rm{per}}\,{\rm{axis}},{\rm{in}}\,{{\rm{m}}}^{2}\end{array}$$

Tree leaf area was calculated as:

4 $$\begin{array}{c}{\rm{Tree}}\,{\rm{leaf}}\,{\rm{area}}=\sum ({\rm{foliated}}\,{\rm{length}}\,{\rm{of}}\,{\rm{all}}\,{\rm{long}}\,{\rm{axes}}\,{\rm{of}}\,{\rm{the}}\,{\rm{tree}})\times {{\rm{C}}}_{{\rm{Long}}}\\ \,\,+\,({\rm{Number}}\,{\rm{of}}\,{\rm{foliated}}\,{\rm{short}}\,{\rm{axes}}\,{\rm{of}}\,{\rm{the}}\,{\rm{tree}})\times {{\rm{C}}}_{{\rm{Short}}},{\rm{in}}\,{{\rm{m}}}^{2}\end{array}$$

This leaf area estimation was considered valid from the start of data acquisition (10/07/2020) until the day just before trimming (22/07/2020). During trimming, the length of all cut parts of the tree axes was measured and a coefficient of leaf area per unit length of these cut parts of the axes was also evaluated with the same method. Thus, the tree leaf area after trimming could be evaluated as the difference of the leaf area before trimming and the leaf area removed by trimming. Since tree aerial development is usually stopped for 2 weeks after a trimming procedure, the tree leaf area can be considered as stable from the day just after trimming (24/07/2020) until 04/08/2020, two weeks later.

The Leaf Area Index (LAI) was calculated for each tree alignment (modality A and modality B) as:

5 $$\begin{array}{c}LAI=\frac{{\rm{Mean}}\,{\rm{Leaf}}\,{\rm{Area}}\,{\rm{per}}\,{\rm{tree}}\times {\rm{number}}\,{\rm{of}}\,{\rm{trees}}\,{\rm{in}}\,{\rm{the}}\,{\rm{alignment}}}{{\rm{crown}}\,{\rm{width}}\times {\rm{crown}}\,{\rm{length}}\,{\rm{of}}\,{\rm{the}}\,{\rm{full}}\,{\rm{tree}}\,{\rm{alignment}}}\end{array}$$

The Leaf Area Density (LAD) was calculated as:

6 $$\begin{array}{c}LAD=\frac{{\rm{M}}{\rm{e}}{\rm{a}}{\rm{n}}\,{\rm{L}}{\rm{e}}{\rm{a}}{\rm{f}}\,{\rm{A}}{\rm{r}}{\rm{e}}{\rm{a}}\,{\rm{p}}{\rm{e}}{\rm{r}}\,{\rm{t}}{\rm{r}}{\rm{e}}{\rm{e}}\times {\rm{n}}{\rm{u}}{\rm{m}}{\rm{b}}{\rm{e}}{\rm{r}}\,{\rm{o}}{\rm{f}}\,{\rm{t}}{\rm{r}}{\rm{e}}{\rm{e}}{\rm{s}}\,{\rm{i}}{\rm{n}}\,{\rm{t}}{\rm{h}}{\rm{e}}\,{\rm{a}}{\rm{l}}{\rm{i}}{\rm{g}}{\rm{n}}{\rm{m}}{\rm{e}}{\rm{n}}{\rm{t}}}{{\rm{c}}{\rm{r}}{\rm{o}}{\rm{w}}{\rm{n}}\,{\rm{w}}{\rm{i}}{\rm{d}}{\rm{t}}{\rm{h}}\times {\rm{c}}{\rm{r}}{\rm{o}}{\rm{w}}{\rm{n}}\,{\rm{l}}{\rm{e}}{\rm{n}}{\rm{g}}{\rm{t}}{\rm{h}}\times {\rm{c}}{\rm{r}}{\rm{o}}{\rm{w}}{\rm{n}}\,{\rm{h}}{\rm{e}}{\rm{i}}{\rm{g}}{\rm{h}}{\rm{t}}\,{\rm{o}}{\rm{f}}\,{\rm{t}}{\rm{h}}{\rm{e}}\,{\rm{f}}{\rm{u}}{\rm{l}}{\rm{l}}\,{\rm{t}}{\rm{r}}{\rm{e}}{\rm{e}}\,{\rm{a}}{\rm{l}}{\rm{i}}{\rm{g}}{\rm{n}}{\rm{m}}{\rm{e}}{\rm{n}}{\rm{t}}}\end{array}$$

The LAI and LAD calculated here are global crown values for each tree alignment: the evaluation procedure employed here does not allow to study spatial variation of leaf area density within the tree crown, like variation with height, for example. In these formulae, the mean leaf area per tree is averaged over the 3 central trees of each alignment, and multiplied by 5, the number of trees in each alignment, to be consistent with the number of trees over which the total crown dimension of the alignment is evaluated.

**Fig. 9:**
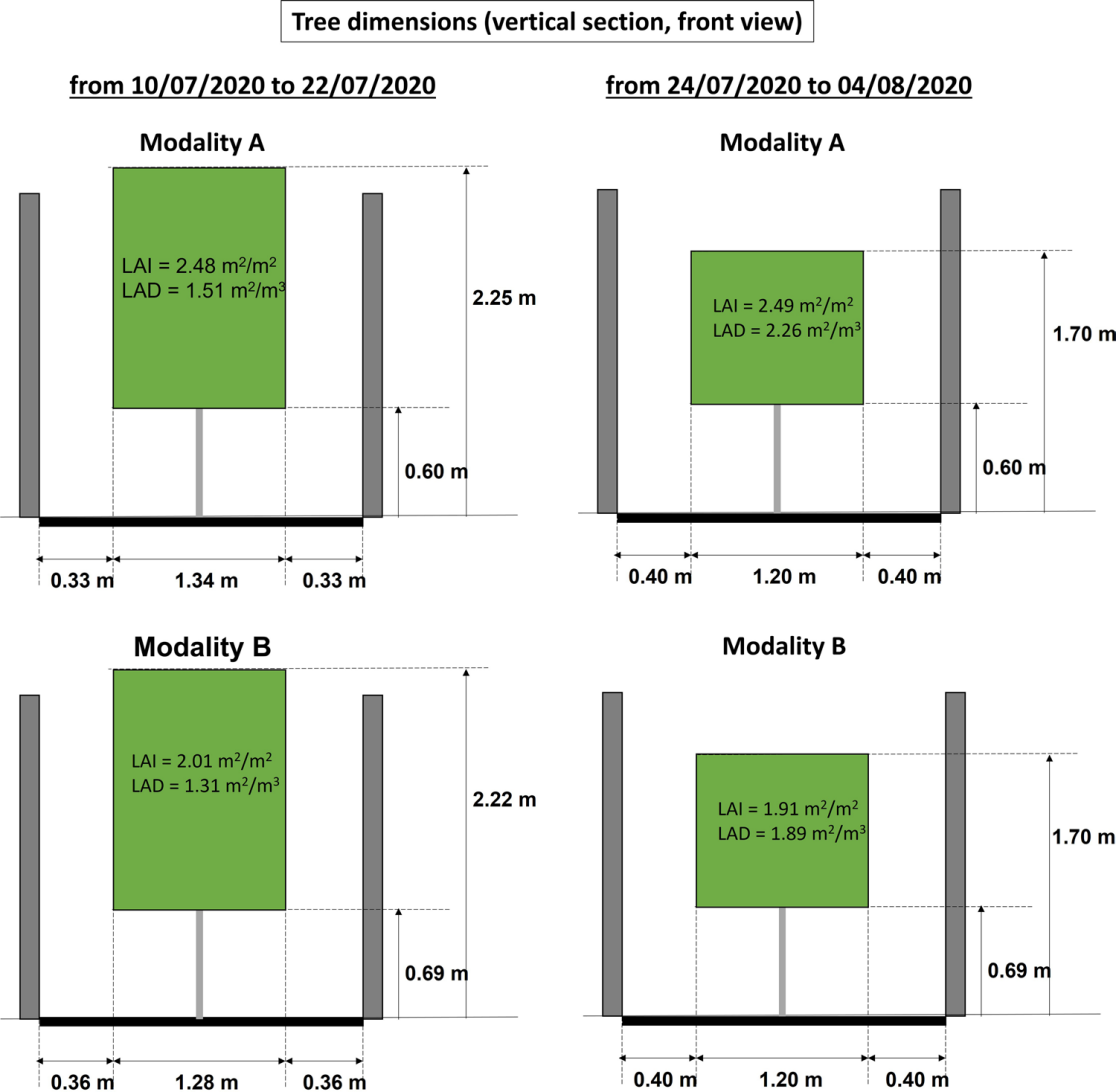
Tree dimensions in the vertical plane (front view), as well as LAI (Leaf Area Index) and LAD (Leaf Area Density), over the two time periods (left, right), for trees in modality A (top) and in modality B (bottom).

**Fig. 10:**
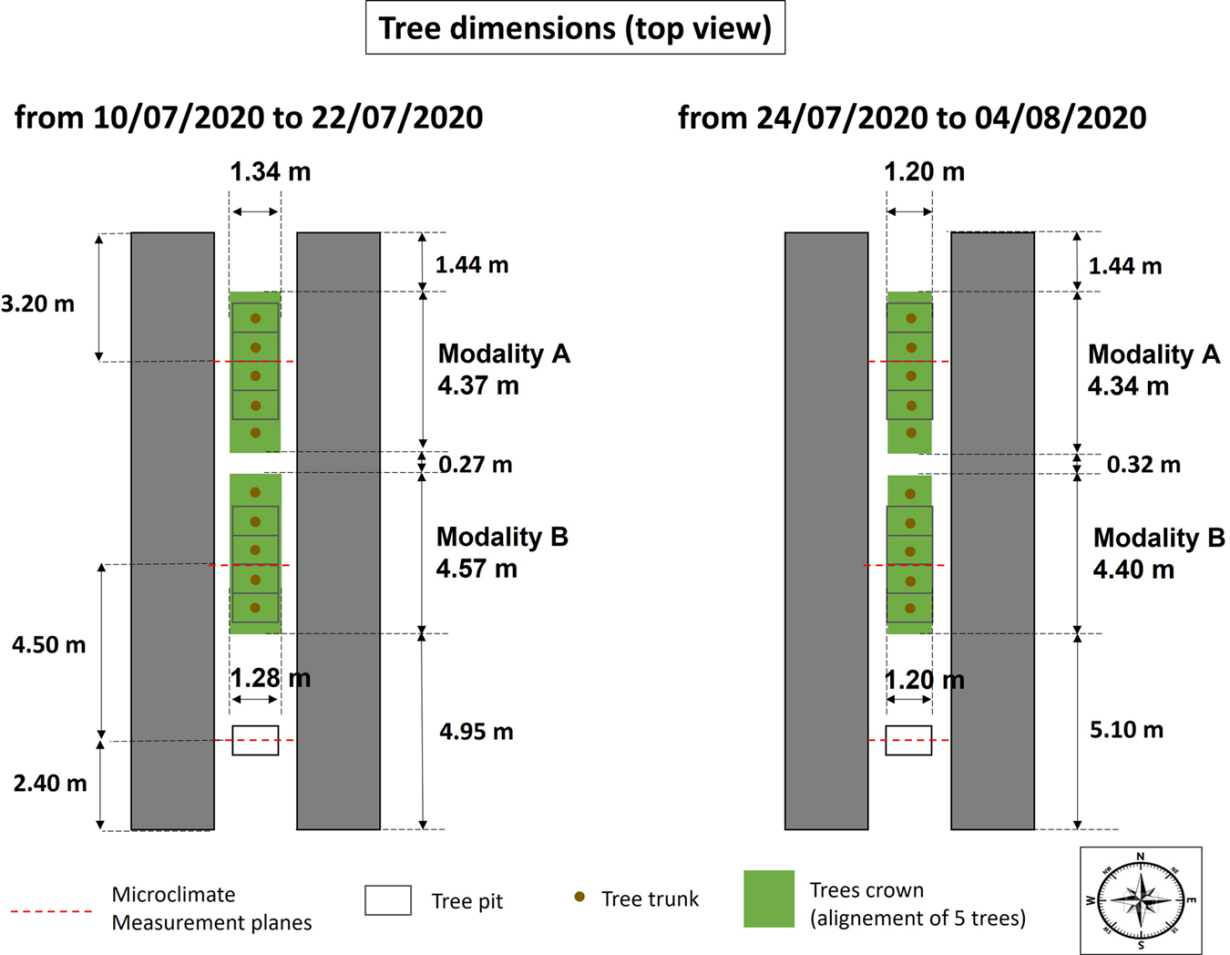
Horizontal extent of each tree alignment.

The values obtained for each modality (A and B) over the two considered sub-periods (before trimming from 10/07/2020 until 22/07/2020, and after trimming, from 24/07/2020 until 04/08/2020), are summarized in Fig. 9 (in a vertical section of each tree), and in Fig. 10 (for the horizontal extent of each tree alignment).

### Tree transpiration and on-site reference evapotranspiration

The methodology used to compute daily tree transpiration is based on a water balance realized on each tree container^13^. The soil water balance can be written as follows in volume of water per day:

7 $$\begin{array}{c}{\rm{P}}+{\rm{I}}-{\rm{D}}-{\rm{ET}}\pm {\rm{R}}=\Delta {\rm{S}}\end{array}$$

With P the total rainfall, I the volume of water brought through irrigation, D the water lost by drainage, ET the evapotranspiration (equal to the sum of evaporation from the soil and transpiration by the tree), R the run-off and ΔS the variation of water content in the soil.

In the present study, a number of simplifications applies: first, the water inputs through rainfall (P) is considered to be negligible, because the pits are covered with a waterproof lid; for the same reason, direct evaporation from the soil is neglected (besides the presence of the lid, the soil is also covered with a mulch layer): the evapotranspiration term (ET) thus reduces to tree transpiration (T); then, there is no slope on the street and there is no direct contact between the border of the pit and the tree container, the run-off (R) is therefore zero.

The water balance equation then reduces to:

8 $$\begin{array}{c}{\rm{I}}\,-\,{\rm{D}}\,-\,{\rm{T}}=\Delta {\rm{S}}\end{array}$$

**Fig. 11:**
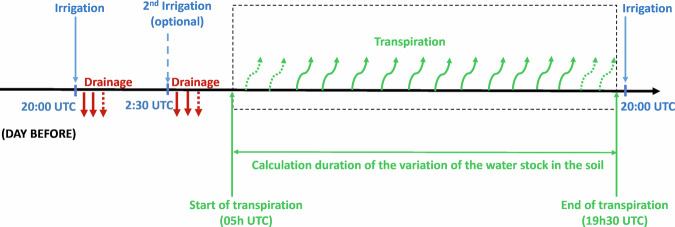
Water balance used for the calculation of daily tree transpiration.

Irrigation was supplied everyday through drippers at 20 h UTC, and optionally with a second irrigation on the next day at 2:30 UTC, with drainage occurring and being collected in the next hours and evaluated in the next morning. Irrigation doses are provided for each day in the dataset. Because there is some uncertainty in the evaluation of drainage (due to possible leak between the drainage pipe and the container, or drainage water exceeding the capacity of the water drainage tank), the water balance was not realized on a 24 h basis, but only during daily transpiration period, once drainage stopped, from 5 h UTC to 19 h:30 UTC everyday (see Fig. 11).

Over this period, there are no irrigation nor drainage fluxes, and the water balance thus reduces to:

9 $$\begin{array}{c}{\rm{Transpiration}}=-\Delta {\rm{S}}=-\Delta \theta \times {{\rm{V}}}_{{\rm{soil}}}\end{array}$$

Transpiration can thus be estimated from the variation of water content in the soil between 5 h UTC and 19 h:30 UTC, given by the variation of volumetric water content (Δθ) multiplied by the volume of soil in the container (V_soil_ = 44.5 L). The variation of volumetric water content is obtained by averaging the variation measured by the two capacitive sensors at 0.22 m depth for each container (Fig. 7). When only one of the two sensors of a given container was working correctly, transpiration was not computed, as it is estimated that it should be computed and averaged on at least two sensors to be representative of water variation in the container. In the water balance, the inputs from rain are neglected because the containers are covered with a lid, as already stated. However, the containers are not fully rainproof, and thus, specific attention was given to the days when rain occurred during the water-balances hours. This was the case on the 14/07/2020, 17/07/2020, 25/07/2020, 27/05/2020, and 03/08/2020 with respectively 2.5 mm/day, 0.2 mm/day, 0.4 mm/day, and 2.2 mm/day of rain measured from Météo France Beaucouzé weather station. The tensiometers and volumetric water content sensors were checked to detect any water infiltration from rain on these days. A small infiltration was detected by tensiometers only on the 14/07/2020, and transpiration values were therefore invalidated (replaced by NAN value) on that day.

For reference on-site daily evapotranspiration (ET_0_), it was evaluated following FAO-56^35^ Penman Monteith equation with the variables measured at 2 m above ground level outside the street, on the west mast (for wind) and on the north mast (for net radiation, air temperature and relative humidity).

### Thermal comfort index: UTCI

The Universal Thermal Climate Index (UTCI) is a human thermal comfort index, based on Fiala’s^36^ thermoregulation model. A simplified calculation of this index makes use of a 6^th^ order polynomial approximation^37^ based on air temperature, relative humidity and mean radiant temperature at human height, and wind velocity at 10 m height (which corresponds to a standard measurement height for wind in meteorological conventions).

In our research facility, the UTCI was calculated at 0.4 m height from the ground, which corresponds to 2 m height at full scale, and is relevant for the evaluation of human thermal comfort. Accordingly, UTCI in each modality was computed at 0.4 m height using air temperature, relative humidity and globe temperature measured at this height in the corresponding modality (see Fig. 5). Mean radiant temperature (T_mrt_) was derived from globe temperature, with a correction for convective heat loss^38^ (Eq. 10):

10 $$\begin{array}{c}{T}_{mrt}=\sqrt[4]{{({T}_{g}+273.15)}^{4}+\frac{1.06\times {10}^{8}\times {V}_{a}^{0.58}}{\varepsilon \times {D}^{0.42}}\times ({T}_{g}-{T}_{a})}-273.15\end{array}$$

With T_mrt_ (°C), T_g_ (°C), and T_a_(°C), the mean radiant temperature, globe temperature, and air temperature respectively, V_a_ (m/s) the wind velocity, D (m) the globe diameter (equal to 0.15 m), and ε the globe emissivity (taken as 0.95). Wind velocity was available at only one position in the street, between modality B and C (see Fig. 5). Therefore, the same wind velocity was used to compute the correction of T_mrt_ for convective heat loss in modalities A, B and C, assuming that at 0.4 m height, which is strictly below tree crown, there is little variation of average wind velocity between vegetated and non-vegetated modalities.

Finally, following UTCI computation guidelines^37^, the wind velocity at 10 m height was derived from the wind velocity measurement at 0.4 m height between modalities B and C, by inverting the logarithmic law of velocity profile that is subsequently used in UTCI calculation algorithm^37^ to compute wind velocity at human height from measurement at 10 m height.

## Data Records

**Fig. 12:**
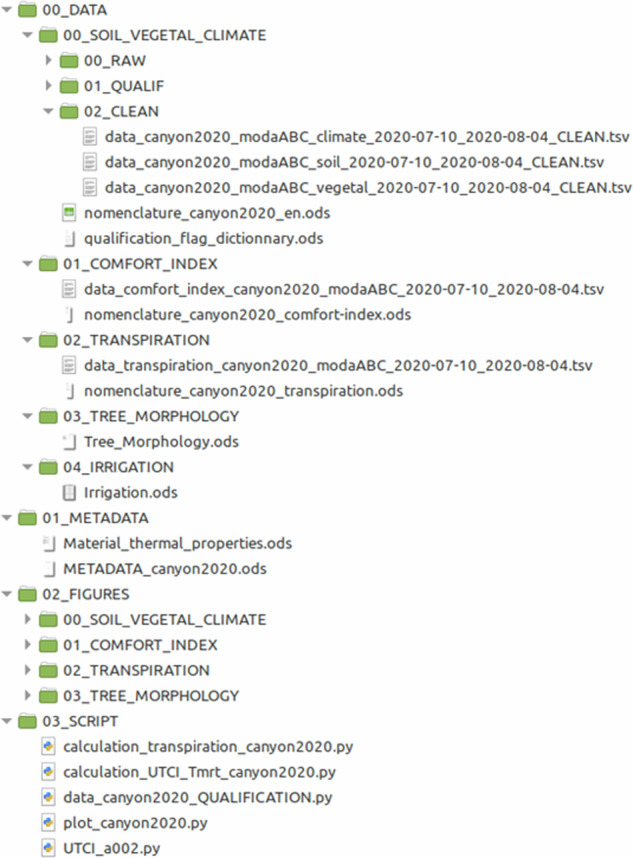
Structure of the dataset, with subfolders.

The dataset^39^ is provided as a zip file along with a readme.txt file, available on Earth System Data Repository (10.57932/29a2ba8b-87e8-4a5d-aba6-ebf6a8faa2c6). The zip file organization is shown in Fig. 12: it is organized in four folders: 00_DATA for the datasets, 01_METADATA for metadata, 02_FIGURES for figures, and 03_SCRIPTS with python scripts used to generate the data and figures.

In the data folder 00_DATA, the data is again organized in several subfolders, depending on variable type:

- 00_SOIL_VEGETAL_CLIMATE contains data in tsv format (tab separated values: values in a text file with tab separator) on microclimate, soil water status and tree-environment interactions, with 10 minutes time step. Three versions of datasets are provided in RAW, QUALIF, and CLEAN subfolders, corresponding respectively to raw data, qualified data, and cleaned data (see next section for more details on the data qualification).
- 01_COMFORT_INDEX contains data with mean radiant temperature and UTCI in tsv format, with 10 minutes time step.
- 02_TRANSPIRATION contains daily tree transpiration data in tsv format
- 03_TREE_MORPHOLOGY contains a spreadsheet file (ods format) with information on tree crown volume, tree leaf area, LAI and LAD
- 04_IRRIGATION contains a spreadsheet file (ods format) with daily water inputs and irrigation time.

**Fig. 13:**
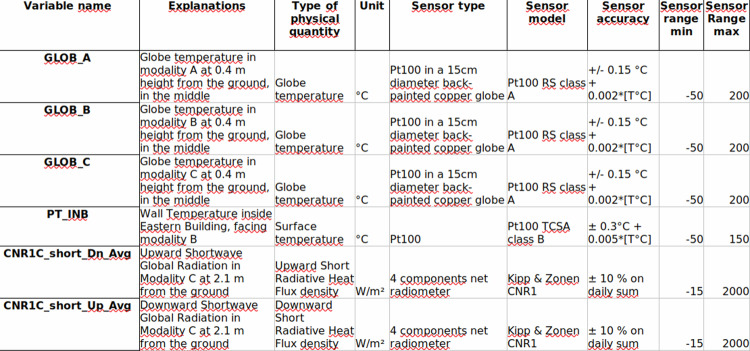
Extract of the nomenclature file “nomenclature_canyon2020_en.ods”. The full nomenclature is available in the dataset.

In each soil vegetal-climate, comfort index, and transpiration datasets, the units are given in the first line, the variable name on the second line, and the data starts on the third line with tab separated values. In each subfolder, a nomenclature spreadsheet (ods format) is provided with variable name, description, unit, and, if appropriate, sensor information (see Fig. 13 for an extract of nomenclature for microclimate variables). As it can be seen in the nomenclature file, all temperature data are in °C, all relative humidity data are in %, all radiation fluxes are in W/m^2^, velocities are in m/s, matric water potential are in hPa, soil volumetric water content are in fraction, tree transpiration are in L/day.

In the metadata folder (01_METADATA) all generic metadata information (GPS location, street dimension, building and tree description…) can be found in file “METADATA_canyon2020.ods”, together with some photos and figures illustrating the experimental facility. A dedicated file contains material thermal properties (“Material_thermal_properties.ods”), when available.

In the 02_FIGURES folder, time evolution of all variables in the CLEAN data file, as well as computed variables (mean radiant temperature, UTCI and transpiration) are provided as pdf image file, in order to get a quick comprehension of the dataset.

In the 03_SCRIPTS folder, custom python scripts written and used for data qualification, computation of comfort index and tree transpiration, and for reading and plotting of the data are provided under MIT licence. A fourth script “UTCI_a002.py” is provided with a python translation of the polynomial approximation for UTCI calculation originally written in fortan90^37^.

## Technical Validation

### Quality assurance

Before installation, all climatic sensors had been calibrated in-house by the manufacturer, and soil volumetric water content (VWC) sensors were calibrated inside the lab in the same soil mixture as the one in the tree containers.

During the measurement period, routine checks were realized every working day on the experimental site in order to visually detect any issue on the facility or on the climatic sensors. A particular attention was paid to surface sensors (leaf temperature sensors, ground and wall temperature sensors) to check that they were well attached to the surface, to the functioning of the fan of the mechanically ventilated TRH sensor, and to cleanliness of the radiation sensors.

Regarding daily irrigation, a control beaker was used to make sure automated irrigation with drippers did work with the expected water input, and drainage water was also collected, to make sure the trees did get enough water for well-watered condition and to re-adjust the irrigation dose if necessary. The accuracy of the irrigation drippers was checked on the 09/07/2020, and a mean difference of −2.6% was found with respect to the water volume set point. Finally, it was also checked every two weeks that tensiometers were still filled with water.

If any problem was detected at this stage, repairing was undertaken and the problem was registered in the laboratory notebook for a posteriori flagging in the quality control procedure.

### Quality control

**Fig. 14:**
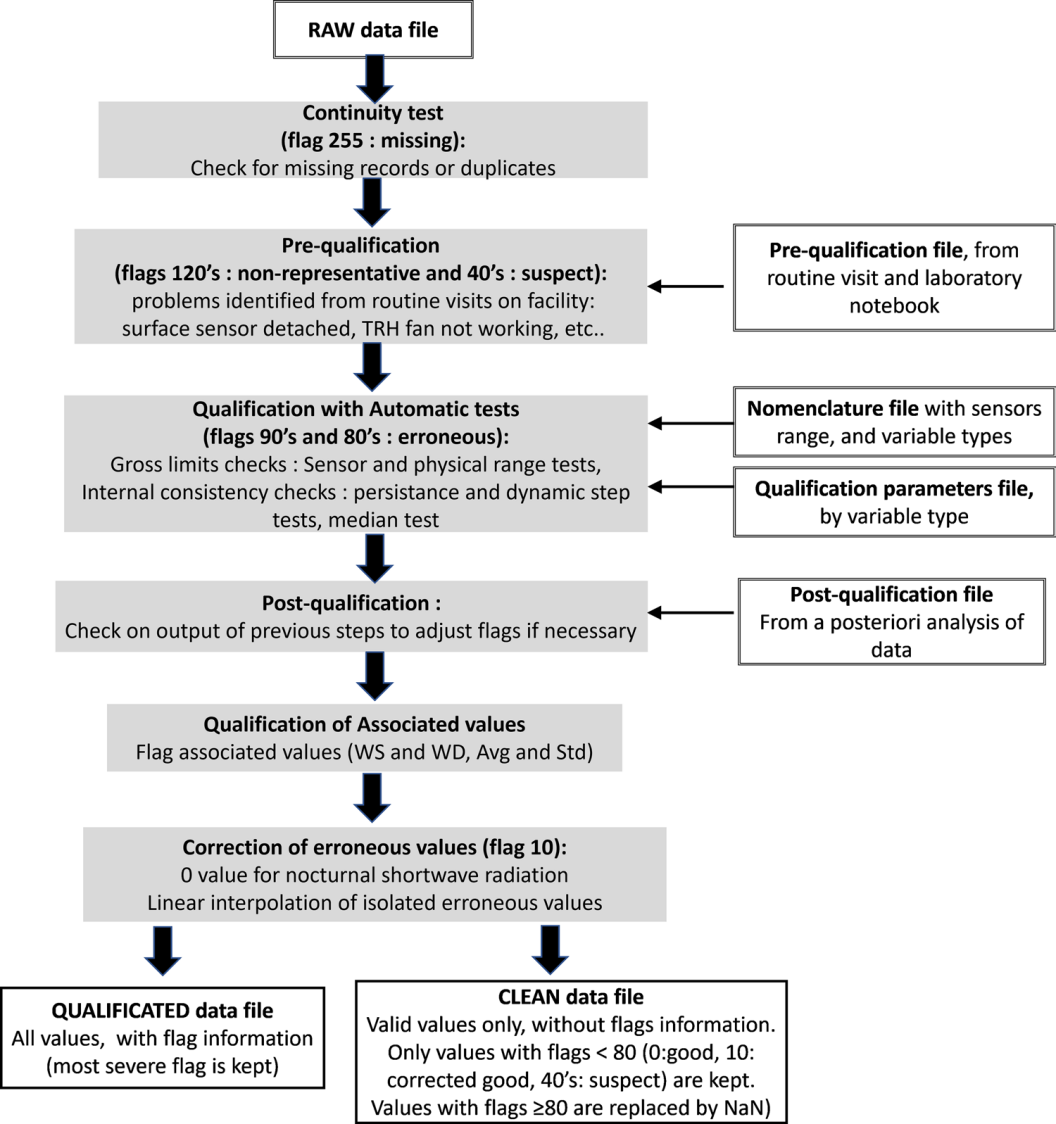
Overview of the quality control procedure.

The quality control procedure is adapted from recommendation of WMO guide^40^ and other references^41–44^. The procedure is described in Fig. 14, and is applied to the microclimate, soil and vegetal raw data files in the folder 00_DATA/00_SOIL_VEGETAL_CLIMATE/00_RAW. The quality control procedure results in two output datafiles for each variable type (microclimate, soil and vegetal data):

- a QUALIFIED datafile, meant for expert users, where each data value is associated to a quality flag, in the folder 00_DATA/00_SOIL_VEGETAL_CLIMATE/01_QUALIF
- a CLEAN datafile, meant for non-expert users, where only values considered as valid (flag < 80) are kept, the non-valid values being replaced by NANs. This file does not contain flag information, and is located in the folder 00_DATA/00_SOIL_VEGETAL_CLIMATE/02_CLEAN

The quality control procedure consists of:

- a continuity test, to identify missing values or duplicate records,
- a pre-qualification step, with problems identified on the field from routine visits and registered in the laboratory notebook,
- an automatic qualification step on the dataset including gross limit checks (sensor range test, physical range test) and internal consistency checks (persistence test, dynamic test, median test),
- a post-qualification step, to verify the output of previous steps from a posteriori analysis of the data, and to adjust the flags if necessary,
- a correction step on erroneous data, if possible.

The flag dictionary, with explanations on the quality control performed, is provided in Table 5. The procedure is written in Python 3, and the scripts files are provided in the data repository, together with the parameter files.

**Table 5:** Flag dictionary, with explanations on the quality control performed.

Flags	Signification	Auto or manual Flagging	Type of variables applied to	Description
**0**	Good	Both	All	Data passed the quality control procedure, without correction.
**10**	Corrected good	Auto	All	Isolated erroneous values are replaced by linearly interpolated values from neighbouring good data.
				All nocturnal short-wave radiation heat flux values that are negative, but lower in absolute value than sensor net irradiance offset value are replaced by zero value.
**40**	suspect	Manual	All	Generic suspect
**41**	Suspect, human activity in the street	Manual	All	Intense human activity in the street (tree trimming, tree measurements…) that may impact the environment and the measurements by sensors.
**42**	Suspect, TRH fan may be off	Manual	AT, HR	Records comprised between last visit when it was OK, and first visit where it was not OK: it is uncertain during this period when the problem started (see code 122)
**43**	Suspect, sensor may not in right place	Manual	All	Same as above for sensor position (see code 123)
**44**	Suspect, radiation sensor may not horizontal	Manual	Vertical radiation	Same as above for radiation sensor horizontality.
**80**	Erroneous		All	Generic erroneous
**81**	Erroneous, failed Persistance Test	Auto	All	Measured value remains unchanged beyond maximum plausible duration (max. duration for each variable type indicated in qualification parameters file)
**82**	Erroneous, failed dynamic Test	Auto	AT, HR, WS	Change from previous and/or next record is above p75 + factor*iqr or below p25-factor*iqr (p75 and p25 are the 75^th^ and 25th percentile respectively, iqr is the interquartile range, and “factor” is indicated in qualification parameters file)
**83**	Erroneous, failed Median Test	Auto	AT, HR	Absolute difference between record and median of all records for this variable type is above a given threshold (threshold based on experience and indicated in qualification parameters file)
**91**	Erroneous, failed Sensor range Test	Auto	All	Measured value if out of sensor manufacturer working range (range given in data nomenclature)
**92**	Erroneous, failed Physical range Test	Auto	All	Measured value if out of plausible physical range: min/max for each variable type derived for 30-years climatic record at Meteo France weather station and user experience (range indicated in qualification parameters file)
**120**	Non-representative		All	Generic non-representative
**121**	Non-representative, radiation sensor artificially shaded	Manual	Radiation sensors	The sensor is subjected to undesired artificial shading from the experimental apparatus itself, or from human operator (from routine visits and/or manual data inspection)
**122**	Non-representative, TRH fan is off	Manual	AT, HR	Routine visits showed that TRH fan is off: records comprised between first visit where fan was NOK, and time when fan was repaired or replaced.
**123**	Non-representative, sensor not in right place	Manual	All	Sensor is not in right place, or fallen off, or surface sensor is no more attached to the surface (from routine visits checks)…
**124**	Non-representative, radiation sensor not horizontal	Manual	Vertical radiation	The radiation sensor is not anymore horizontal (from routine visits checks)
**255**	Missing data	Auto	All	Data is missing

AT: Air Temperature; RH: relative humidity; WS: windspeed (velocity magnitude).

## Usage Notes

For microclimate, soil and vegetal data, it is advised to non-expert data users to use the CLEAN files available in the folder 00_DATA/00_SOIL_VEGETAL_CLIMATE/02_CLEAN. All units are provided in the first line of the datasets, or in the nomenclature file nomenclature_canyon2020_en.ods.

The users can have a quick look at the CLEAN data with figures available in the folder 02_FIGURES.

If the datasets are used to validate microclimate models, it is recommended to use the following measurements and variables in the microclimate datafile “data_canyon2020_modaABC_climate_2020-07-10_2020-08-04_CLEAN.tsv” as boundary conditions for the numerical simulations:

- for incident radiation heat fluxes: measurements from radiometer CNR1 at 2.1 m above ground level in modality C: variables “CNR1C_short_Up_Avg” for incident downward shortwave radiation, and “CNR1C_lg_UpCo_Avg” for incident downward longwave radiation, respectively.
- For incident wind speed and direction: wind measurement from anemometers on the west mast outside the street: “WS10m_S_WVT” and “WD10m_D1_WVT” for wind speed and wind direction at 10 m from the ground, “Wind_Speed_avg5m” and “Wind_Dir_avg5m” for wind speed and direction at 5 m from the ground, and “Wind_Speed_avg2m” and “Wind_Dir_avg2m” for wind speed and direction at 2 m from the ground, respectively.
- For incident temperature and relative humidity: measurement from TRH sensor at 2 m from the ground on the North mast outside the street: variables “TaV_2M_North_Avg” and “RHV_2m_North_Avg” for air temperature and relative humidity respectively.

The users should recall and note that the data inside the street is acquired in a reduced-scale, north-south oriented street, with an aspect ratio of 1 and a central row of trees, in a suburban environment. It can be used for the evaluation of numerical models, for this type of configuration, provided the spatial resolution of the model is fine enough for the scale of the street.

The dataset should be interpreted with caution when trying to transpose present results to an *in-situ* full scale urban configuration. A first discussion on the topic of representativity of the experimental site and data can be found in a previous article^13^.

Radiation data in our reduced-scale canyon street (radiative heat fluxes, surface temperatures differences, globe temperature differences) depend on incident radiation, shape factors and thermal characteristics of materials and can therefore be considered as representative of full-scale measurements, because here geometrical similarity is ensured and urban materials are representative of a full-scale environment, with similar aspect ratio and street orientation.

In contrast, air temperature, air humidity and wind can be affected by atmospheric mixing with the surrounding environment. For example, air temperature differences measured between the non-vegetated and vegetated modalities of the street cannot be readily interpreted as the air-temperature difference that would be obtained between a vegetated and a non-vegetated zone in a dense city, due to the difference of scale and surrounding environment.

## Data Availability

The custom scripts in Python 3 used for data qualification, and computation of tree transpiration, as well as thermal comfort indexes are provided with the dataset, under MIT licence.
